# Reading Between the Striatal Lines: Magnetic Resonance Imaging Insights Into Huntington Disease

**DOI:** 10.31486/toj.25.0052

**Published:** 2025

**Authors:** Robert Boothe, David J. Houghton, James Milburn

**Affiliations:** ^1^ Department of Radiology, Ochsner Clinic Foundation, New Orleans, LA; ^2^ Department of Neurology, Ochsner Clinic Foundation, New Orleans, LA; ^3^ The University of Queensland Medical School, Ochsner Clinical School, New Orleans, LA

## TO THE EDITOR

Huntington disease (HD) is a rare neurologic disease with a poor prognosis. To optimize management for patients and caregivers, a multidisciplinary team that includes movement disorder specialists, genetic counselors, social workers, physical and occupational therapists, speech therapists, psychologists, and palliative care experts is crucial. To assist patients with finding appropriate care, the Huntington's Disease Society of America (HDSA) has designated HD centers of excellence throughout the United States. Ochsner Health has been recognized for many years as an HDSA center of excellence to help close the care gap in the Gulf South. The Ochsner Health HD program, led by David J. Houghton, MD, has dedicated HD clinics twice monthly where patients meet with each member of the multidisciplinary team to identify any care needs. This approach ensures that patients and caregivers receive the necessary medical, social, and emotional support in a single visit.

In this letter, we highlight the genetic, clinical, and neuroradiologic features of HD to familiarize readers with the disease. We also provide treatment and resource information.

## HUNTINGTON DISEASE BACKGROUND

HD, or Huntington chorea, is an autosomal dominant neurodegenerative condition that causes progressive neurologic and psychiatric symptoms. Prevalence varies throughout different geographic populations, with an estimated incidence of 10 to 14 individuals per 100,000 in western countries and only rare cases in Asian and African populations.^1,2^ HD can affect any age group. Juvenile HD presents in individuals younger than 20 years and accounts for approximately 5% to 10% of patients with HD.^3^ While patients with juvenile HD develop similar motor, cognitive, and psychiatric symptoms as adults, juvenile HD is characterized by speech and language delays, seizures, and a more rapid overall decline compared to adults.^3^ Prognosis is poor in juvenile HD, with death often occurring within 7 to 8 years of diagnosis, compared to 15 to 18 years in adults.^3,4^

One famous example of a patient with HD is American singer/songwriter Woodie Guthrie who lost his ability to speak and hold a guitar and died at age 55.^5^

The HTT gene is found on chromosome 4 and contains 6 to 35 trinucleotide repeats of cytosine-adenine-guanine in the normal population.^2,6^ An aberrant amount of more than 40 trinucleotide repeats causes full penetrance, meaning patients with these repeats will develop HD in their lifetimes.^1,2^ These trinucleotide repeats encode the huntingtin protein found throughout the body; however, the neuropathology of HD is specific to the striatum of the basal ganglia.^2^ With expansion of the gene, a mutant form of the protein is produced, leading to neurologic dysfunction (eg, cognitive deficits and abnormal movements).^1^

Dr George Huntington brought HD to medical attention in 1872. In Dr Huntington's paper, he describes the chorea as a third decade disease with classic presentation of chorea progressing to eventual spastic movements of all extremities.^7^ Since Dr Huntington's early characterization of the disease, medical advancements such as genetic testing and neuroimaging have led to improved characterization. The mainstay of HD diagnosis is genetic analysis and early clinical recognition of disease presentation.

## RADIOGRAPHIC APPEARANCE

While structural differences in the brain can be seen on computed tomography (CT), magnetic resonance imaging (MRI) has superior spatial resolution that allows for greater soft tissue detail when visualizing structures in the brain. Common imaging characteristics of HD in the juvenile and adult forms include bilateral atrophic changes in the caudate head and putamen.^4,8^ These changes can result in enlargement of the frontal horns of the lateral ventricles, forming a characteristic boxcar configuration.^9^ Age-advanced generalized cerebral volume loss is also a feature of HD.^10^ Compared to CT, MRI provides improved resolution of caudate head atrophy, as well as of signal changes in both the caudate head and putamen.^8^

**Figure 1.**
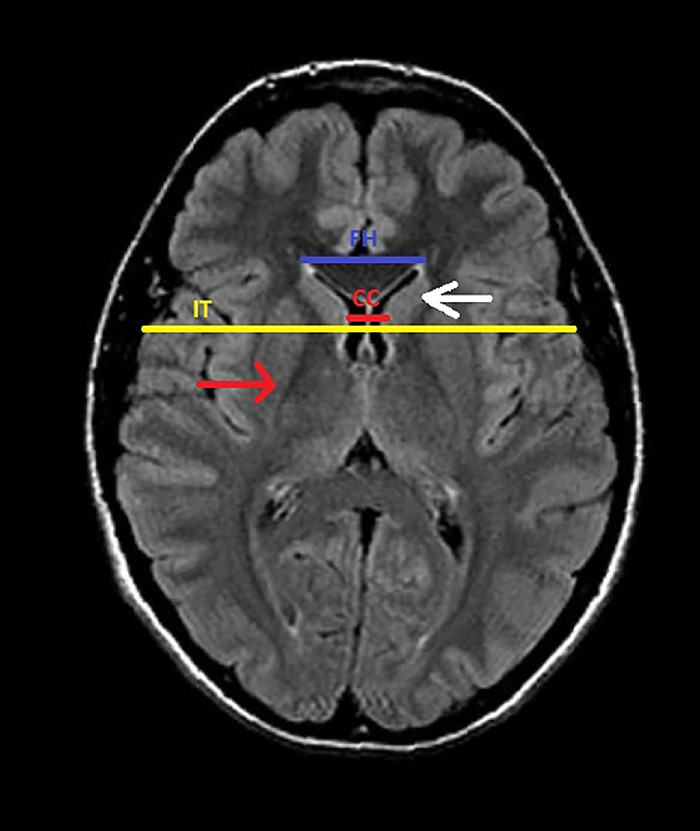
Axial T2-FLAIR (fluid-attenuated inversion recovery) magnetic resonance image of a normal brain shows the structures affected in Huntington disease and the measurements used to calculate the frontal horn width to intercaudate distance (FH/CC) ratio and the intercaudate distance to inner table width (CC/IT) ratio: caudate head (white arrow), putamen (red arrow), frontal horn (FH) width (blue line), intercaudate (CC) distance (red line), and inner table (IT) width (yellow line).

While exact measurements may not be specific for a diagnosis, measurement techniques such as the frontal horn width to intercaudate distance (FH/CC) ratio and the intercaudate distance to inner table width (CC/IT) ratio are a standardized method of evaluating caudate head atrophy.^11,12^ The FH/CC ratio is normally 2.2 to 2.6; however, as caudate head volume decreases, the ratio approaches 1. The CC/IT ratio is normally 0.09 to 0.12; the ratio conversely increases as caudate head volume decreases.^4^ Figure 1 shows the measurements used to calculate the ratios and demonstrates the normal anatomic appearance of the caudate head and putamen.

To illustrate the radiographic appearance of HD, we present 3 representative cases of patients with HD. Each patient had atrophy of the bilateral caudate heads and putamina with associated enlargement of the frontal horns of the lateral ventricles.

**Figure 2.**
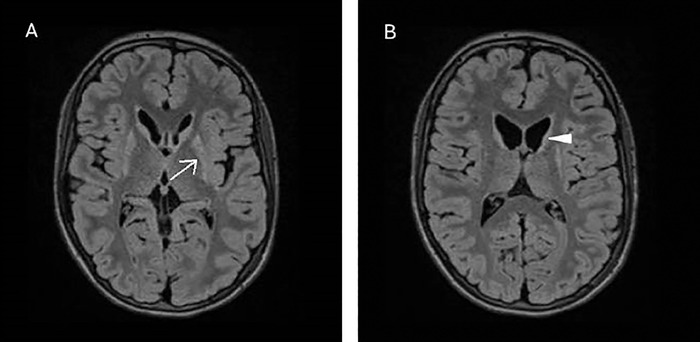
3D SPACE (sampling perfection with application-optimized contrasts using different flip angle evolutions) FLAIR (fluid-attenuated inversion recovery) magnetic resonance images of the brain of Case 1 show atrophy of the bilateral caudate heads and putamina, with associated signal increase in the putamina. The left putamen is identified with a white arrow in image A. The left caudate head is identified with a white arrowhead in image B.

Case 1 is a 21-year-old female who was diagnosed with juvenile HD when she was 9 years old. The patient previously attended school but because of disruptive behavior was transitioned to home schooling. The patient's father had HD. This patient had increased signal in the putamen (Figure 2), an imaging characteristic seen in juvenile HD.^4^ The FH/CC ratio was reduced at 1.4. The CC/IT ratio was elevated at 0.22. Both ratios quantify the visualized caudate head atrophy that coincides with the patient's disease state.

Cases 2 and 3 are adult patients. In addition to atrophic changes in the caudate head and putamen, the 2 adult patients had age-advanced generalized cerebral volume loss. While cerebral volume loss is a nonspecific finding, it is typically seen in patients with HD.^10^ In both adult cases, the ratios quantify caudate head atrophy.

**Figure 3.**
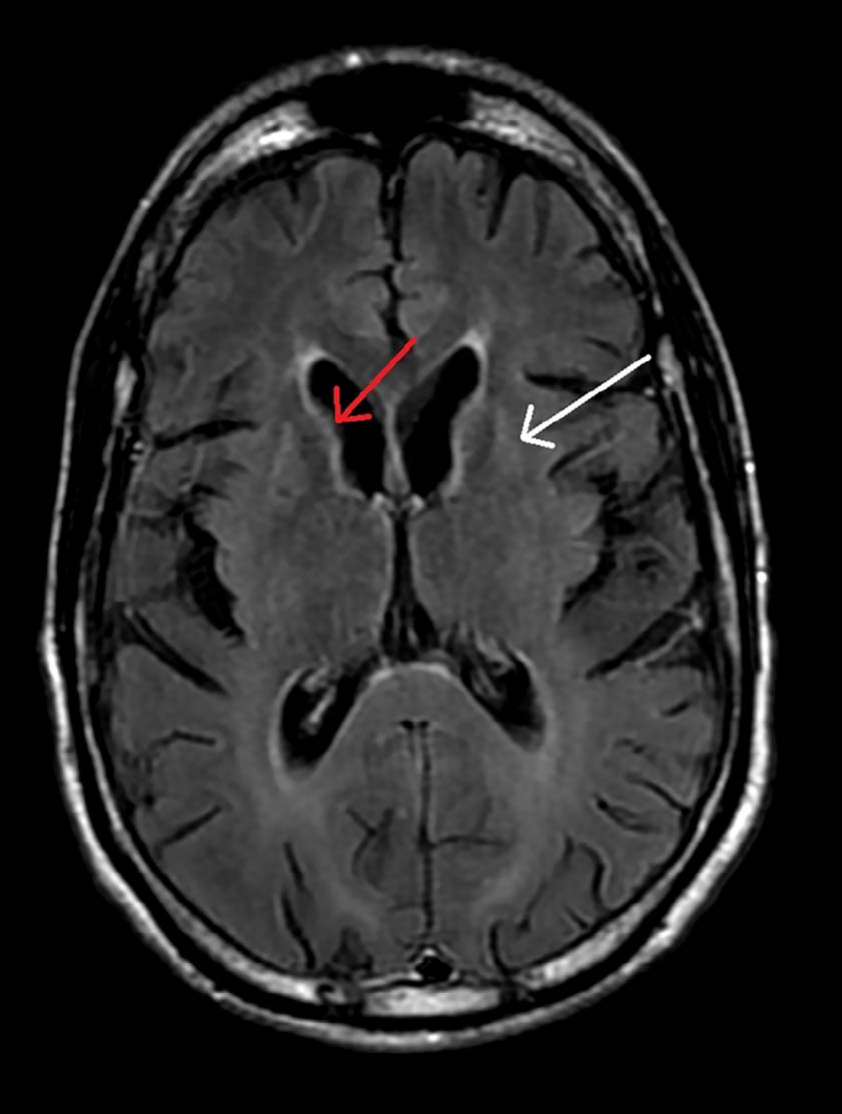
Axial T2-FLAIR (fluid-attenuated inversion recovery) magnetic resonance image of the brain of Case 2 shows severe volume loss in the caudate heads and putamina bilaterally. The right caudate head is indicated with a red arrow. The left putamen is indicated with a white arrow.

Case 2 is a 53-year-old male who initially noticed issues with balance and coordination and later began to experience dysphagia with liquids and solids. He was initially diagnosed with parkinsonism, but various medical treatments offered no improvement. Approximately 5 years after symptom onset, he was diagnosed with HD. The patient had a history of depression and methamphetamine use. He had been adopted, but he located his birth mother and learned that she did not know of anyone in her family with similar symptoms. The identity of the birth father was unknown to the patient and his birth mother. In this patient, the FH/CC ratio was reduced at 1.8, and the CC/IT ratio was elevated at 0.18 (Figure 3).

**Figure 4.**
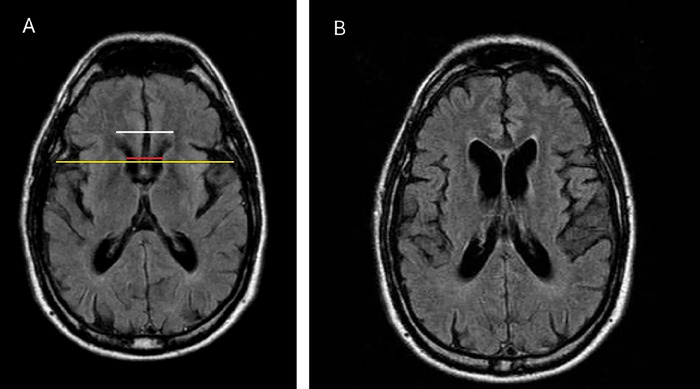
Axial T2-FLAIR (fluid-attenuated inversion recovery) magnetic resonance (MR) images of the brain of Case 3 show 2 slices through the brain (images A and B) demonstrating moderate diffuse cerebral atrophy with decreased volume of the bilateral caudate heads. Annotations in image A show how the frontal horn (FH) width (white line), the intercaudate (CC) distance (red line), and the inner table (IT) width (yellow line) are measured on axial MR images. Measurements are as follows: FH=36.2 mm, CC=22.7 mm, and IT=115 mm. These measurements are used to calculate a reduced FH/CC ratio of 1.6 and an elevated CC/IT ratio of 0.20.

Case 3 is a 54-year-old male with a history of HD in his mother and brother. Given the known family history, the patient was diagnosed in early adulthood via genetic testing. The timing of symptom onset is unknown; however, his symptoms initially manifested as choreic movements. As the disease progressed, the patient also began to experience bradykinesia and manic episodes. In this patient, the FH/CC ratio was reduced at 1.6, and the CC/IT ratio was elevated at 0.20 (Figure 4).

## TREATMENT AND RESOURCES

Treatment for HD is symptomatic and focused on supportive care, although research trials investigating potential disease-modifying treatments are underway. The HDSA maintains an up-to-date record of US Food and Drug Administration–approved medications for symptomatic treatment in HD, as well as a list of target therapies currently being investigated.^13^ HD Trialfinder is another resource from the HDSA that patients, caregivers, physicians, and healthy volunteers can use to find and potentially join ongoing clinical trials.^14^ When possible, patients with HD should be referred to a specialized HD clinic so they receive the highest quality support and disease management.^15^
